# Real-world experience of ocrelizumab in multiple sclerosis patients in Latin America

**DOI:** 10.1590/0004-282X-ANP-2020-0339

**Published:** 2021-05-08

**Authors:** Juan Ignacio ROJAS, Liliana PATRUCCO, Manuel FRUNS, Giesela HORNUNG, José FLORES, Edgar CARNERO CONTENTTI, Pablo Adrian LOPEZ, Juan Pablo PETTINICCHI, Alejandro CARIDE, Lorna GALLEGUILLOS, Jorge BARAHONA, Violeta DIAZ, Marianella HERNÁNDEZ, Ricardo ALONSO, Edgardo CRISTIANO

**Affiliations:** 1 Hospital Universitario de CEMIC, Servicio de Neurología, Buenos Aires, Argentina. Hospital Universitario de CEMIC Servicio de Neurología Buenos Aires Argentina; 2 Centro de Esclerosis Múltiple de Buenos Aires, Buenos Aires, Argentina. Centro de Esclerosis Múltiple de Buenos Aires Buenos Aires Argentina; 3 Hospital Italiano de Buenos Aires, Servicio de Neurología, Buenos Aires, Argentina. Hospital Italiano de Buenos Aires Servicio de Neurología Buenos Aires Argentina; 4 Clínica las Condes, Santiago de Chile, Chile. Clínica las Condes Clínica las Condes Santiago de Chile Chile; 5 Instituto Nacional de Neurología y Neurocirugía, Ciudad de México, México. Instituto Nacional de Neurología y Neurocirugía Ciudad de México México; 6 Centro Neurológico ABC Santa Fé, Ciudad de México, México. Centro Neurológico ABC Santa Fé Ciudad de México México; 7 Hospital Alemán, Department of Neuroscience, Neuroimmunology Unit, Buenos Aires, Argentina. Hospital Alemán Department of Neuroscience Neuroimmunology Unit Buenos Aires Argentina; 8 Clínica Alemana de Santiago, Unidad de Neuroinmunología, Santiago, Región Metropolitana, Chile. Clínica Alemana de Santiago Unidad de Neuroinmunología Santiago, Región Metropolitana Chile; 9 Hospital Ramos Mejía, Centro Universitario de Esclerosis Múltiple, Buenos Aires, Argentina. Hospital Ramos Mejía Centro Universitario de Esclerosis Múltiple Buenos Aires Argentina; 10 Hospital Universitario Sanatorio Guemes, Servicio de Neurología, Buenos Aires, Argentina. Hospital Universitario Sanatorio Guemes Servicio de Neurología Buenos Aires Argentina

**Keywords:** Multiple Sclerosis, Pharmaceutical Preparations, Latin America, Effective Life, Esclerosis Múltiple, Preparaciones Farmacéuticas, América Latina, Vida Efectiva

## Abstract

**Background:**

Despite the abundance of information concerning ocrelizumab in phase III clinical trials, there is scarce evidence regarding real-world patient profiles.

**Objective:**

The aim of this study was to investigate patient profiles, effectiveness and persistence with treatment among patients who used ocrelizumab for treatment of multiple sclerosis in Latin America.

**Methods:**

This was a retrospective multicenter study in Argentina, Chile and Mexico. Medical record databases on patients who received ocrelizumab were analyzed. Demographic and clinical variables were described, along with effectiveness outcomes, which included the proportions of patients free from clinical relapses, from disability progression and from new or enlarging T2 or T1 gadolinium-enhancing lesions, on annual magnetic resonance imaging.

**Results:**

A total of 81 patients were included. The most frequent phenotype was relapsing-remitting MS, in 64.2% of the patients. The mean age at study entry was 41.3 ± 12.0 years and 51.8% were women. A total of 38% had had relapse activity during the 12 months before starting on ocrelizumab, with a mean relapse rate of 1.3 ± 0.6 during that period. 75% were free from clinical relapses and 91% were free from gadolinium-enhancing lesions in the relapsing-remitting course. Ocrelizumab discontinuation during the first 12 months was observed in three patients (3.7%). The mean persistence observed during the first-year follow-up was 338 ± 24 days.

**Conclusions:**

Our study is in line with previous randomized clinical trials and recent real-world studies describing patient profiles, effectiveness and persistence regarding ocrelizumab treatment in multiple sclerosis patients in Latin America.

## RESUMEN

**Introducción:**

A pesar de la abundante información sobre ocrelizumab proveniente de los ensayos clínicos de fase III, todavía se tiene poca evidencia sobre la efectividad y el perfil de pacientes provenientes de la vida real.

**Objetivo:**

Evaluar el perfil clínico y demográfico, la efectividad y la persistencia al tratamiento en pacientes que usaron el ocrelizumab para el tratamiento de esclerosis múltiple (EM) en Latinoamérica.

**Métodos:**

Estudio retrospectivo multicéntrico en Argentina, Chile y México. Se analizaron los datos de los pacientes que recibieron ocrelizumab. Se describieron las variables demográficas y clínicas, así como los resultados de efectividad que incluyeron la proporción de pacientes libres de recaídas clínicas, libres de progresión de la discapacidad, libres de nuevas lesiones en la secuencia T2 o T1 con gadolinio durante el seguimiento.

**Resultados:**

Se incluyeron 81 pacientes. El fenotipo más frecuente fue EM remitente recurrente (EMRR) en el 64,2% de los pacientes. La edad media fue de 41.3±12 años, y el 51,8% eran mujeres. Un total de 38% tuvo recaídas durante los 12 meses previos al inicio de ocrelizumab, con una tasa anualizada de recaídas media de 1.3±0.6 durante ese período. En el seguimiento a 12 meses, el 75% estuvo libre de recaídas clínicas y el 91%, libre de nuevas lesiones en RM. Tres pacientes interrumpieron el tratamiento durante el seguimiento (3,7%). La persistencia al tratamiento observada durante el primer año de seguimiento fue de 338±24 días.

**Conclusión:**

Nuestro estudio está en línea con los datos provenientes de ensayos clínicos aleatorizados previos y estudios recientes del mundo real que describen la efectividad de los perfiles de pacientes y la persistencia al tratamiento con ocrelizumab en pacientes con EM en Latinoamérica.

## INTRODUCTION

Multiple sclerosis (MS) is a chronic inflammatory disease of the CNS that leads to focal plaques of primary demyelination and diffuse neurodegeneration in the grey and white matter of the brain and spinal cord^1^. In most patients, the disease starts with a relapsing-remitting course (RRMS), which is followed for several years by a secondary progressive phase (SPMS). Patients with primary progressive disease (PPMS) skip the relapsing and remitting stage and start with uninterrupted progression from disease onset^2^.

It has been almost 25 years since the publication of the pivotal trial results for the first disease-modifying therapy (DMT) for RRMS^3^. Currently, the DMTs for MS that have been approved by the European Medicines Agency (EMA) and the Food and Drug Administration (FDA) include interferon beta (IFNβ) 1­a and 1­b, glatiramer acetate (GA), mitoxantrone, natalizumab, fingolimod, teriflunomide, dimethyl fumarate, alemtuzumab and ocrelizumab^4^.

Ocrelizumab was approved in March 2017 for the treatment of relapsing or primary progressive MS^5^. A phase II trial established 600 mg intravenously every 6 months as the preferred dosing schedule. Two phase III trials evaluated the efficacy of ocrelizumab in patients with RRMS and individual and pooled analyses demonstrated significant reductions in the annualized relapse rate (p < 0.001 pooled), disability progression at 12 weeks (p < 0.001 pooled) and gadolinium-enhancing lesions on magnetic resonance imaging (MRI; p < 0.001)^5^. Patients with PPMS were evaluated in a third phase III trial, which showed a significant decrease in both disease progression at 12 weeks (p = 0.03) and the volume of T2-weighted lesions on MRI (p < 0.001)^5^. As with other monoclonal antibodies, the adverse effects seen with ocrelizumab were primarily infusion-related reactions and infection^5^. Despite the abundance of information concerning the efficacy and safety of ocrelizumab in phase III clinical trials, there is scarce evidence regarding real-world patient profiles.

The aim of this study was therefore to evaluate patient profiles, effectiveness and persistence with treatment during follow-up, in a retrospective study on patients who were prescribed ocrelizumab for treatment of MS in Latin America (LATAM).

## METHODS

We conducted a retrospective multicenter study in Argentina, Chile and Mexico. We reviewed all medical record databases of patients who received ocrelizumab and were followed for at least one year before and after starting treatment. Only patients with a diagnosis of MS defined according to validated criteria were considered for inclusion in the study^6^^,^^7^.

### Clinical parameters evaluated at baseline

The demographic and clinical characteristics of the disease were collected at the time when use of ocrelizumab was started. Age and gender data were extracted, along with disease characteristics including the following: age at onset, disease duration since the first relapse (defined as detection of the first sign/symptom that suggested CNS demyelination in the optic nerves, brain stem, spinal cord or other regions and which was not attributable to other diseases^8^), clinical and radiological activity during the year previous to ocrelizumab treatment (clinical activity defined as new relapse and radiological activity, such as new T2 or GAD MRI lesions), number of relapses, EDSS score (pre-treatment), prior exposure to DMTs and reasons for change of treatment to ocrelizumab.

### Follow-up evaluation

Once ocrelizumab had been started, the patients were followed for at least 12 months for the analysis. Clinical evaluations every three months tended to collect information about the following three matters: a) Clinical relapses. These were defined as the appearance of a new neurological symptom that lasted more than 24 hours, in the absence of clinical intercurrence, followed by a period of clinical stability or improvement of at least 30 days. b) Progression of physical disability. This was evaluated through clinical evaluation by applying the EDSS scale. This variable was dichotomized for analysis, into patients who progressed in EDSS and patients who did not progress. Progression was defined as a worsening of 1 point on the scale between one measurement and another, with an interval of at least 6 months between them. To consider a case to be one of progression, if there had been a clinical relapse, the patient needed to be 3 months away from the relapse, regardless of whether steroid treatment had been received for management of the acute episode. c) New lesions found on MRI. MRI was done using 1.5 tesla equipment, with slices of thickness 3 to 5 mm. The sequences obtained were T1, T2, FLAIR and T1 with intravenous contrast. MRI scans were obtained at baseline and at 12 months, on each patient.

### Persistence evaluation

Information from the patients was collected for at least 12 months after use of ocrelizumab was started. During the follow-up, the proportion of patients discontinuing the treatment with ocrelizumab over the 12 months after inclusion and the reasons for discontinuation were registered. These reasons were categorized into four groups: 1) poor tolerability, i.e. when discontinuation was ‘patient driven’ due to side effects; 2) adverse events, i.e. when discontinuation was ‘physician-driven’ due to medical concerns regarding expected or unexpected side effects; 3) disease activity, i.e. radiological or clinical events that led physicians to discontinue treatment because of lack of efficacy; and 4) others, i.e. any reason not included in the previous definitions.

### Storage and data analysis

Once patients had been identified and had consented to participate, variables from the patient charts were transferred to the specifically designed web platform, with restricted access by users and a password specific to each researcher. The data shared were anonymized and only demographic and clinical data were accessible. Patient data such as name, surname and ID were not visible to the analysts.

The study was approved by the local ethics committee of each participating center, and written or oral informed consent was obtained from all participants.

### Statistical analysis

Continuous data were expressed with their means and SD. Categorical data were expressed as percentages. Demographic and clinical variables were described, along with the proportion of the patients who discontinued the treatment with ocrelizumab over the 12 months after inclusion. Persistence was a continuous value defined as the number of days from the date of starting ocrelizumab use to the date of discontinuation of the index treatment. Statistical analyses were performed using the Stata 15 software.

## RESULTS

### Study population and baseline characteristics

A total of 81 patients met the inclusion criteria and were included (38.3% were from Argentina, 40.7% from Chile and 21% from Mexico) (Table 1). Many of the patients included were part of the compassionate use of ocrelizumab in Latin America. The most frequent phenotype was RRMS, in 64.2% of the patients included (Table 1). The mean age of the patients at study entry was 41.3 ± 12.0 years, and 51.8% of the patients were women. The mean disease duration was 8.4 years, and most of the patients included were in employment at the time of study entry (77%). The principal characteristics of the patients included are presented in Table 2. The main reason for starting ocrelizumab among RRMS patients was treatment failure, in 48%, while among PPMS patients the most frequent reason was disease progression (defined as EDSS progression).

**Table 1.** Patient distribution according to country and disease phenotype.

	Total	RRMS	PPMS
Argentina, n (%)	31 (38.3)	5 (16.1)	26 (83.9)
Chile, n (%)	33 (40.7)	30 (90.9)	3 (9.1)
Mexico, n (%)	17 (21.0)	17 (100)	0
Total, n (%)	81 (100)	52 (64.2)	29 (35.8)

RRMS: relapsing-remitting multiple sclerosis; PPMS: primary progressive multiple sclerosis.

**Table 2.** Patient characteristics at baseline.

	Total	RRMS	PPMS
Mean age (years) ± SD	41.3 ± 12.0	37.8 ± 12.0	47.4 ± 12.0
Female sex, n (%)	42 (51.8)	31 (60)	11 (38)
Mean EDSS ± SD	3.1 ± 1.8	2.8 ± 1.9	3.6 ± 1.7
Mean disease duration (years) ± SD	8.4 ± 6.3	8.8 ± 7.2	7.8 ± 4.3
Working status			
Employed	63 (77)	40 (77)	23 (79)
Unemployed	18 (23)	12 (23)	6 (21)
Previous DMT, n (%)	62 (76.5)	45 (86)	17 (58)
Type of previous DMT, n (%)			
Beta interferon	23 (37.0)	16 (35.5)	7 (41.0)
Glatiramer acetate	2 (3.2)	2 (4.5)	0
Teriflunomide	1 (1.6)	1 (2.2)	0
Fingolimod	5 (8.0)	2 (4.5)	0
Dimethyl fumarate	1 (1.6)	1 (2.2)	3 (17.7)
Natalizumab	14 (22.5)	11 (24.5)	3 (17.7)
Rituximab	16 (25.8)	12 (26.7)	4 (23.5)
Previous 2 or more DMT, n (%)	41 (50)	32 (61)	9 (31)
Reason for starting use of ocrelizumab			
Treatment failure with previous DMT	20 (25)	25 (48)	6 (21)
Adverse event with previous DMT	31 (38)	14 (27)	6 (21)
Disease progression	30 (37)	13 (25)	17 (58)

RRMS: relapsing-remitting multiple sclerosis; PPMS: primary progressive multiple sclerosis; EDSS: expanded disability status scale; DMT: disease-modifying treatment; SD: standard deviation.

A total of 38% of the patients included had had relapse activity during the 12 months before starting use of ocrelizumab. During that period, the mean relapse rate was 1.3 ± 0.6. Almost all the relapses in the cohort were treated with corticosteroids (96%). EDSS progression was observed in 49.4% of patients during the previous 12 months, while new T2 MRI lesions were described in 68% of the patients. The activity during the 12 months before use of ocrelizumab was started is described in Table 3.

**Table 3.** Patient characteristics before and after starting treatment with ocrelizumab.

	RRMS			PPMS		
	Year before treatment with ocrelizumab	After starting treatment with ocrelizumab	p-value	Year before treatment with ocrelizumab	After starting treatment with ocrelizumab	p-value
Relapse activity, n (%)	32 (62)	8 (15)	0.01	11 (38)	2 (7)	0.37
Mean relapse rate ± SD	1.4 ± 0.7	0.23 ± 0.4	< 0.001	1 ± 0.3	0.22 ± 0.15	0.01
Steroid treatment for relapse, n (%)	30 (95)	3 (37)	0.001	9 (100)	2 (100)	0.67
EDSS progression, n (%)	21 (40.4)	2 (4)	0.31	19 (65.5)	9 (31)	0.09
GAD + MRI activity, n (%)	30 (58.0)	4 (8)	0.06	10 (34.5)	2 (7)	0.44
T2 MRI activity, n (%)	41 (78.8)	18 (35)	0.001	19 (65.5)	7 (24)	0.05

RRMS: relapsing-remitting multiple sclerosis; PPMS: primary progressive multiple sclerosis; SD: standard deviation; GAD, MRI and T2: types of lesions.

### Effectiveness

Among RRMS patients, during the follow-up, 15% had a relapse, 4% progressed in EDSS and 8% had new gadolinium lesions on follow-up MRI (Table 3). Among PPMS patients, 7% had a relapse, 31% progressed in EDSS and only 2 patients had a new gadolinium lesion on MRI during follow-up (Table 3). Among both RRMS and PPMS patients, there was a reduction in the annualized relapse rate, in comparison with the year before ocrelizumab treatment was started (1.4 ± 0.7 vs. 0.23 ± 0.4; p < 0.001; and 1 ± 0.3 vs. 0.22 ± 0.15; p = 0.01; in RRMS and PPMS respectively) (Table 3).

### Persistence evaluation

**Figure 1.**
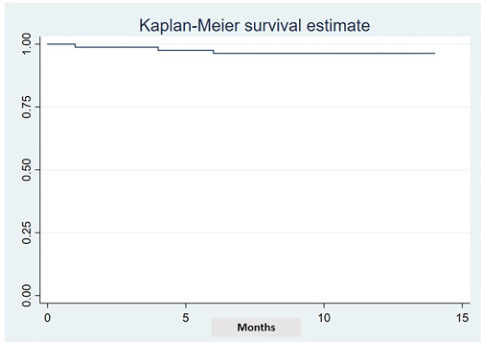
Persistence with ocrelizumab treatment during the study period.

Regarding ocrelizumab administration and persistence during the first year of ocrelizumab treatment, the mean time between the first administration (300 mg) and the second administration (corresponding to the first cycle) was 16 days, while the period between the first and the second cycles was 6.1 months. Ocrelizumab discontinuation during the first 12 months was observed in 3 patients (3.7%). The reasons are described in Table 4. The mean persistence observed at the time of the first-year follow-up was 338 ± 24 days (Figure 1).

**Table 4.** Persistence with ocrelizumab use during the 12 months of follow-up.

	Total	RRMS	PPMS
Mean number of ocrelizumab cycles, n ± SD	3.6 ± 0.62	3.6 ± 0.5	3.5 ± 0.82
Days between first and second applications, mean ± SD	16.0 ± 2.6	16.3 ± 2.9	16.8 ± 3.1
Months between first and second cycles, mean ± SD	6.1 ± 0.6	6.1 ± 0.7	6.2 ± 0.77
Months between second and third cycles, mean ± SD	6.5 ± 0.84	6.5 ± 0.81	6.6 ± 0.91
Months between third and fourth cycles, mean ± SD	7.1 ± 0.9	6.4 ± 0.8	7.3 ± 0.95
Ocrelizumab discontinuation, n (%)	3 (3.7)	1 (1.9)	2 (6.7)
Reason for discontinuation			
Poor tolerability	1 (33.3)	0	1 (50)
Disease activity	1 (33.3)	0	1 (50)
Other	1 (33.3)	1 (100)	0

RRMS: relapsing-remitting multiple sclerosis; PPMS: primary progressive multiple sclerosis; SD: standard deviation.

## DISCUSSION

The aim of this study was to describe patient profiles, effectiveness and persistence regarding ocrelizumab treatment in MS patients in Latin America. We analyzed 81 patients at three centers in Argentina, two centers in Chile and one center in Mexico. The most frequent phenotype was RRMS, in 64.2% of the patients included. The main reason for starting use of ocrelizumab among RRMS patients was treatment failure (in 48%), while among PPMS patients the most frequent reason was disease progression (defined as EDSS progression). EDSS progression had been observed in 49.4% of the patients during the previous 12 months, while new T2 MRI lesions were described in 68% of the patients. Among both RRMS and PPMS patients, there were reductions in the annualized relapse rate, comparing the year before and the year after ocrelizumab treatment was started. Ocrelizumab discontinuation during the first 12 months was observed in 3 patients, while 96.3% of the patients were continuing the treatment after 12 months of follow-up.

The results from our study are in line with previous randomized clinical trials and recent real-world studies. In OPERA I, OPERA II and ORATORIO, the patient profiles for RRMS and PPMS were not different from those described in our study, in terms of age, gender distribution and disease duration. Regarding adverse events leading to treatment discontinuation during 96 weeks of follow-up, these were observed in 3.2% of the patients in OPERA I, while in OPERA II discontinuation was observed in 3.8% of the patients in the ocrelizumab arm. This rate was similar to the frequency observed in our study^9^. In the ORATORIO trial, the proportion of the patients in the ocrelizumab arm who discontinued the treatment due to adverse events was reported to be 4.1% after two years of follow-up^10^.

Our study has certain limitations. One important weakness was the low number of patients recruited. Although a greater number of patients could have given a different power to the study, our number permitted the intended analysis. Another limitation was the observational design and the lack of randomization and control group. Lastly, there was only a short follow-up (up to one year).

Our results nevertheless represent the first post-marketing studies conducted in Latin America and in its region, on the use of ocrelizumab in a real-world setting. The importance of this study lies in the possibility that it has provided for exploring other conditions beyond the efficacy and safety of specific treatments, in large populations of patients that are not typically included in initial randomized controlled trials, thereby improving our knowledge about a specific treatment in clinical practice^11^^,^^12^.
